# Clinical utility of SPECT/CT and CT-dacryocystography-enhanced dacryoscintigraphy in the imaging of lacrimal drainage system obstruction

**DOI:** 10.1007/s12149-019-01385-2

**Published:** 2019-07-16

**Authors:** Sandor Barna, Ildiko Garai, Kornel Kukuts, Rudolf Gesztelyi, Laszlo Toth, Adam Kemeny-Beke

**Affiliations:** 1 Scanomed Ltd, Nagyerdei krt. 98, Debrecen, 4032 Hungary; 2 Department of Pharmacology and Pharmacotherapy, Faculty of Medicine, University of Debrecen, Nagyerdei krt. 98, Debrecen, 4032 Hungary; 3 Department of Otorhinolaryngology and Head and Neck Surgery, Faculty of Medicine, University of Debrecen, Nagyerdei krt. 98, Debrecen, 4032 Hungary; 4 Department of Ophthalmology, Faculty of Medicine, University of Debrecen, Nagyerdei krt. 98, Debrecen, 4032 Hungary

**Keywords:** SPECT/CT, Dacryoscintigraphy, Dacryocystography, Nasolacrimal duct obstruction, Epiphora

## Abstract

**Purpose:**

Epiphora is commonly caused by a relative or complete occlusion in the lacrimal drainage system (LDS), principally a nasolacrimal duct obstruction (NLDO). Dacryoscintigraphy (DSG), an extensively assessed imaging technique in diagnosing its abnormalities, can provide only planar images, according to which it needs to be improved. Our aim was to evaluate clinical utility of simultaneous DSG and single-photon emission computed tomography/computed tomography (SPECT/CT) combined with computed tomographic dacryocystography (CT-DCG) in the evaluation of LDS.

**Methods:**

Dynamic imaging with DSG was performed, and tracer radioactivity was detected by a gamma camera. Successively, SPECT/CT images of the involved region were gained, followed by CT-DCG, during which a contrast medium was syringed into the affected LDS, and finally contrast CT scans were obtained again from the same region.

**Results:**

Fifty-seven patients, mean age 54.25 (± 18.26) years all with unilateral NLDO and 32 control subjects, all with patent LDS, mean age 49.88 (± 18.61) years were evaluated in the study. Delayed outflow of tearing eyes was exposed to DSG compared to the fellow and control eyes. The highest value for sensitivity was observed for SPECT/CT, followed by CT-DCG and DSG techniques, while combining DSG with SPECT/CT, DSG with CT-DCG, and SPECT/CT with CT-DCG, the sensitivity increased to 96.49%, 92.98%, and 94.73%, respectively.

**Conclusions:**

Although DSG is a sensitive nuclear medicine method, it only provides useful clinical data when simultaneously supplemented with SPECT/CT and CT-DCG trials as they jointly can offer valuable information about the localization of an abnormality and verify stenosis or obstruction.

## Introduction

Excessive tearing also known as epiphora, a common complaint in ophthalmological practice, especially in the elderly, is due to an imbalance between production and drainage of tears. Several etiologies can provoke an excess of tears [1], and there are various ways to deal with this condition [2–4]. Currently, there is a lack of consensus on managing tearing patients, and it is particularly true for diagnostic and differential diagnostic facilities. Once excluding primary overproduction and reflexive tearing due to secondary overproduction [5], diverse anatomical malformations of the lacrimal drainage system (LDS) permitting tear fluid flow from the eye into the nasal cavity can be encountered. Abnormalities can occur on the one part at the entry of the excretory system, but more often in the lower part of the LDS, of which stenosis or obstruction may be developed by various pathologic reasons, such as congenital malformations, infections of the eye or nose, traumatic disruption, however, most frequently by idiopathic inflammatory obstruction [2]. These disorders are clinically characterized by epiphora, purulent discharge, and more or less frequently by sterile or infected dacryocystitis. An operative solution is indicated in case conservative medical therapy fails. Scantiness of diagnostic utilizations is unambiguously underpinned by a survey carried out among the members of the American Society of Plastic and Reconstructive Surgery revealing that less than 5% of them applied any pre-operative imaging procedure on patients with assumed nasolacrimal duct obstruction (NLDO) [6].

In the evaluation of LDS patency, dacryoscintigraphy or dacryoscintillography (DSG), a readily available, safe, objective, non-invasive, low-radiation-dose nuclear medicine method first introduced by Rossomondo and colleagues [7], and modified by numerous authors [8, 9], is expansively practiced. Even though it is useable not just in assessing LDS patency during diagnostic workup, but also in evaluating tear outflow following a lacrimal surgery, it allows solely planar, two-dimensional images, consequently its sensitivity and specificity need to be improved.

To the best of our knowledge, our working group was the first to report the simultaneous application of DSG and single-photon emission computed tomography/computed tomography (SPECT/CT) combined with computer tomographic dacryocystography (CT-DCG) in the management of NLDO [10], and here we are presenting our extended outcomes dealing with this procedure as a protocol focusing on its effectiveness and clinical utility.

## Patients and methods

### Study population

Consecutive patients who mentioned epiphora as a major complaint were recruited into our prospective, interventional, cross-sectional, case–control study. The diagnosis of NLDO was made based on detailed ophthalmic investigations that included slit lamp examination, tear break-up time (tBUT), and Schirmer tests, syringing to assess LDS patency, as well as otorhinolaryngological endoscopic evaluation of the nasal cavity. An age- and gender-matched population for control was also enrolled in the study during the same period selected from subjects presenting for routine eye examination with minor refractive errors (± 1.0 diopter), and no history of any tearing problem. None of the patients or controls took any medications that may have influenced tear secretion or drainage at the time of the measurements and none of them took any eye drops 2 weeks prior to measurements and during study days. Other exclusion criteria for both groups were abnormal eyelid position and closure, contact lens wearing, history of ocular surgery, presence of ocular infection, inflammation of sclera, episcleral layer and uvea, trauma of the eye, corneal haze, peripheral or central corneal melting.

### DSG technique

**Fig. 1:**
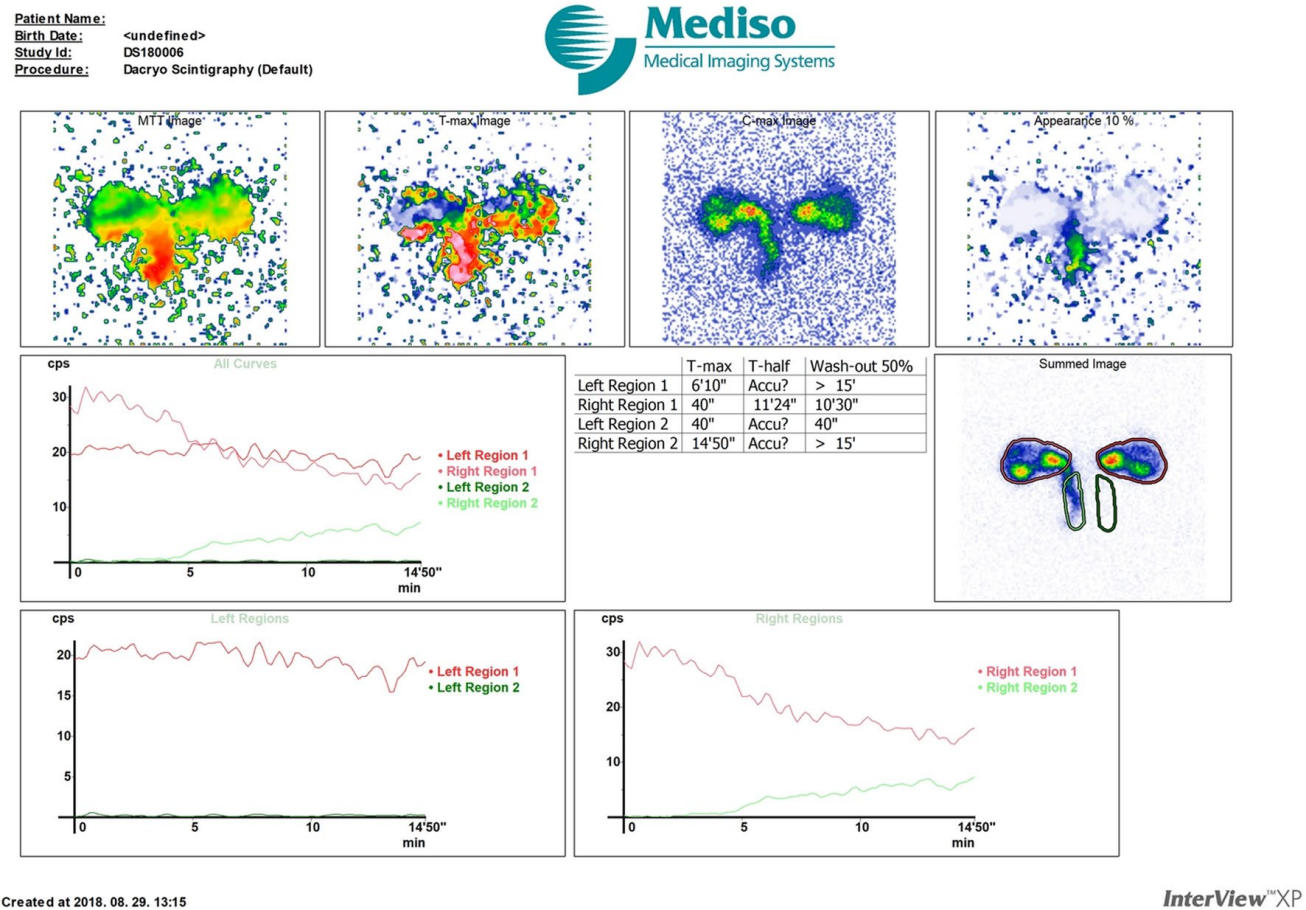
DSG result of a patient suffering from left side NLDO: the right side is patent, while a significant obstruction on the left side stops tear outflow from the region of eye toward the lacrimal pathway. *DSG* dacryoscintigraphy, *NLDO* nasolacrimal duct obstruction, *left region 1* region of the left eye, *right region 1* region of the right eye, *left region 2* region of the left nasolacrimal duct, *right region 2* region of the right nasolacrimal duct

**Fig. 2:**
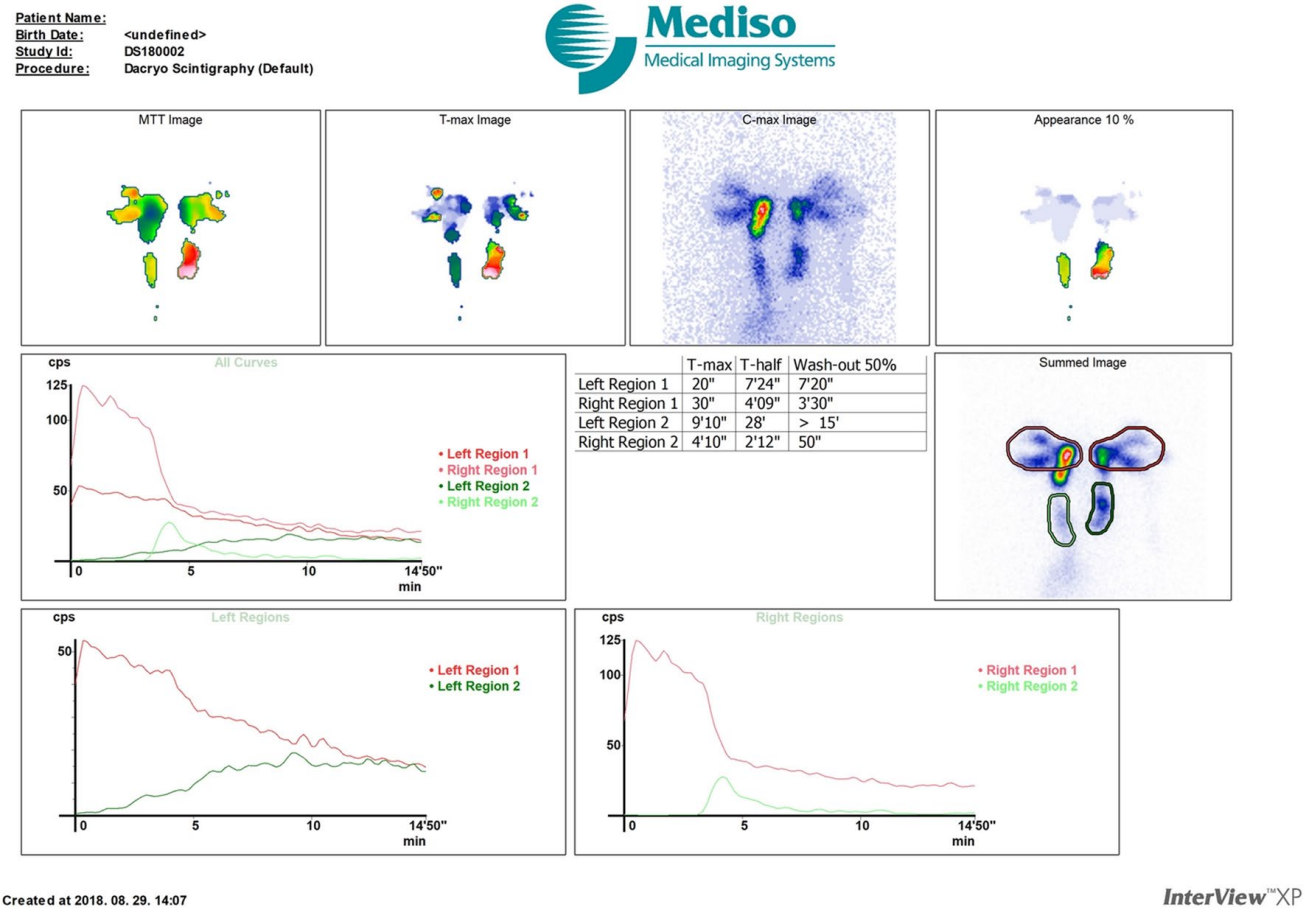
DSG result of a control subject: both sides are patent, for this reason normal scintigrams can be depicted for both sides. *DSG* dacryoscintigraphy, *left region 1* region of the left eye, *right region 1* region of the right eye, *left region 2* region of the left nasolacrimal duct, *right region 2* region of the right nasolacrimal duct

For the DSG process, a massage was applied on both lacrimal sacs to remove any debris out of the upper part of tear drainage system. For dynamic investigation of the regions of the eye and LDS, one drop (20 µL) of 100 MBq/mL concentration, effectively 2 MBq activity of ^99m^Tc-sodium pertechnetate as a radioactive tracer with a half-life for gamma emission of 6.0058 h in a saline solution, was administered per eye with a micropipette without using any local anesthetic eye drop. Special attention was taken to prevent any contamination of the patients’ face with radiolabeled tear, which could interfere with the interpretation of the study. After administration, patients and members of the control group were placed in a sitting position in front of a planar camera for imaging. To ensure patients’ immobilization, their head was secured by a gum strip to obtain optimal imaging. Measurements were performed according to a dynamic data acquisition protocol using 10 s/frame for 15 min (90 × 10 s), which resulted in summed DSG images. Tracer radioactivity as it passes along the lacrimal pathway was detected by a gamma camera (Mediso Nucline TH/22, Budapest, Hungary) with a low-energy high-resolution collimator using a 128 × 128 matrix. After investigations, both eyes were flushed with saline to help clear the remaining radioactivity. Data were also appraised in special regions of interest (ROIs) separately, and consecutive time activity curves were created (Figs. 1, 2). T_max_ and T_½_ (tear clearance) values were calculated based on the activity curves. T_max_ is the time point when the time activity curve reaches the maximum value, while T_1/2_ time is the point on the descending part of the curve, when the time activity curve reaches the moiety of T_max_ activity value.

All DSG results were evaluated by two experienced observers (SB and KK), who were familiar with DSG and unaware of other clinical data, and the final evaluation was made by consensus. Controls were only tested by DSG due to ethical reasons.

### SPECT/CT and CT-DCG examinations in patients

Followed by DSG, a SPECT/CT examination occurred in supine position. During the SPECT investigation, the protocol was as follows: 32 frames per detector head, each with duration of 20 s, 360°, and acquisition matrix size of 128 × 128. The 2D OSEM (ordered subset expectation maximization) reconstruction method was used. A smooth filter and a Butterworth filter were applied with the following parameters: cutoff to zero: 70; gain: 0; cutoff frequency: 0.32; order: 25; no post-filters were implemented.

**Fig. 3:**
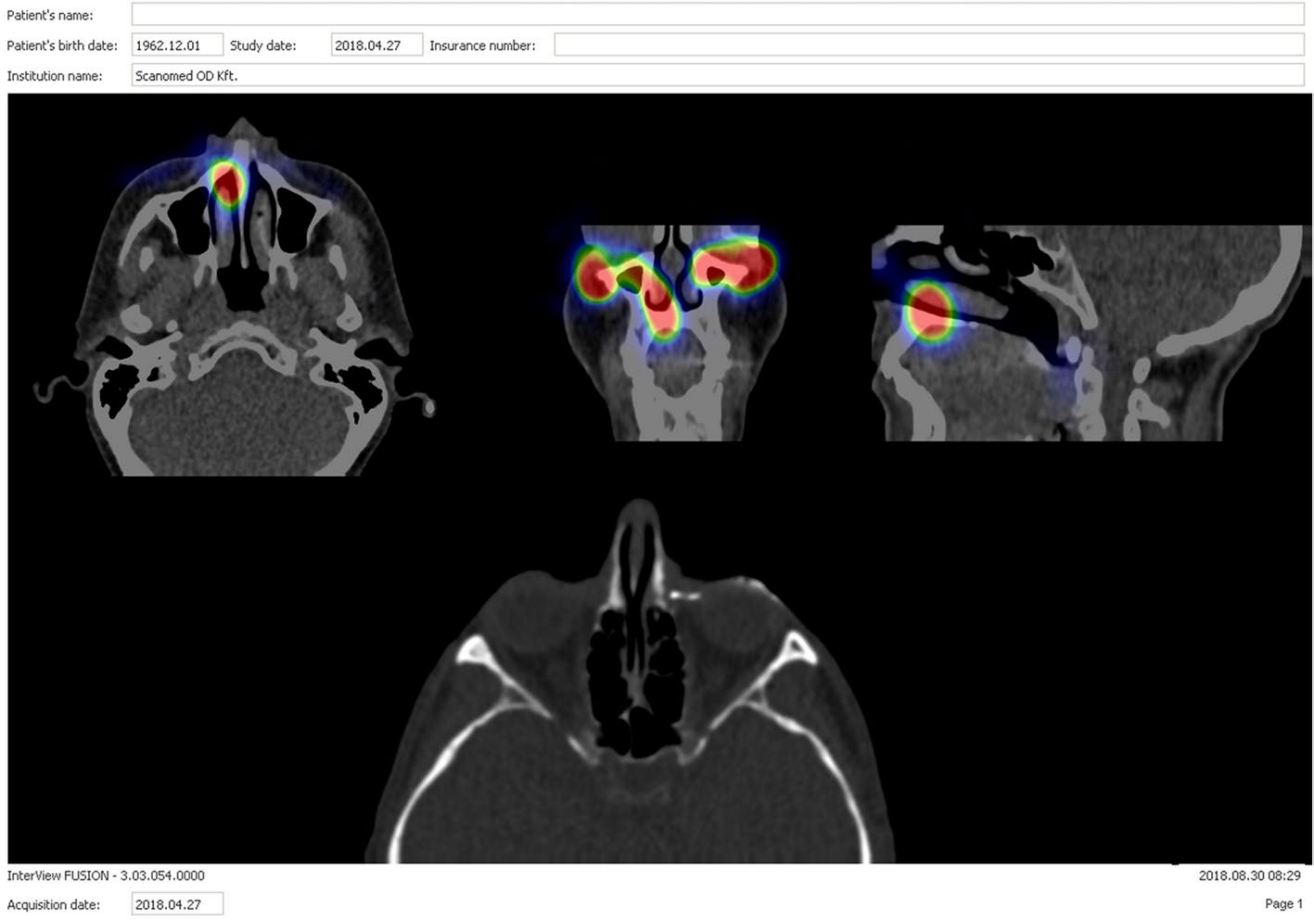
SPECT/CT result of the patient represented in Fig. 1 on coronal and sagittal images: activity of ^99m^Tc-sodium pertechnetate can be detected on the right region of the nasolacrimal duct, but there is no outflow on the left side. *SPECT*/*CT* single-photon emission computed tomography/computed tomography

The CT acquisition was conducted immediately after the SPECT acquisition along these lines: image matrix size: 128 × 128, tube voltage 120 kV, and 50 mAs (AnyScan FLEX SC, Budapest, Hungary). Then followed by locally anesthetizing the conjunctival sac by administering 2% xylocaine eye drops, 0.5–1 mL of contrast material (ioversol, Optiray 350, Guerbet, France) was syringed into the affected LDS by a trained ophthalmologist (A K-B); sequentially contrast CT scans for the same coordinate frame also applying the previous protocol were acquired. Data were obtained as axial images and then reconstructed into 2D and 3D coronal and parasagittal planes along the major axis of the LDS (Fig. 3).

The study protocol was approved by the local ethics committee (Regional and Institutional Research Ethical Committee [DE RKEB/IKEB 4779-2017] together with the National Institute of Pharmacy and Nutrition [OGYEI/37752/2017]) and was in full compliance with good clinical practices (GCP) guidelines of the European Union, and the Declaration of Helsinki (1996). By signing a written informed consent, all patients and controls agreed to have the study results regarding any side effects as well as possible risks and benefits of the study published.

### Statistical analyses

Both eyes of the patients and one randomly selected eye from each control were selected for the statistical analyses. Tracer disappearance times were expressed in seconds as mean values and standard deviations (± SD), whereas categorical variables were expressed as frequencies and their percentages. Distribution of data was checked using the Kolmogorov–Smirnov test. A non-parametric (Wilcoxon) test was used to compare groups. Correlation coefficients between variables were calculated with Spearman’s method. Kappa test was used to compare inter-rater reliability between different methods. *p* values less than 0.05 were considered statistically significant. For the statistical analysis, IBM SPSS 24 statistical software (IBM Corp., Armonk, New York, USA) was used.

## Results

### Characteristics of patients and controls

Fifty-seven consecutive patients, 45 women and 12 men, age 54.25 (± 18.26) years, and 32 control subjects, 24 women and 8 men, age 49.88 (± 18.61) years were examined. All patients were referred with unilateral watering eye and were measured followed by a negative syringing. There was no significant difference between demographic data of patients and controls.

### DSG, SPECT/CT and CT-DCG outcomes

Concerning DSG outcomes, the difference calculated between mean T_1/2_ values in the eye region both for tearing, fellow, and control eyes and mean T_max_ values for tearing and fellow eyes was significant. Interestingly, in the NLD region, mean T_max_ value was the most decreased for the tearing eye with a higher value for the fellow eye, and the highest for the control eye. The difference was statistically also significant. Data are presented in Table 1.

**Table 1:** Maximum (T_max_) and half-life (T_1/2_) tracer and radioactive tracer disappearance time values in the region of eyes and NLD in the tearing, fellow, and control eyes

Mean tracer disappearance time (s)	Tearing eye (*n* = 57)	Fellow eye (*n* = 57)	Control eye (*n* = 32)	*p* tearing eye vs. fellow eye	*p* tearing eye vs. control eye	*p* fellow eye vs. control eye
Eye T_max_	45 ± 56	48 ± 90	37 ± 150	0.794	**< 0.001**	**< 0.001**
Eye T_1/2_	1579 ± 427	1274 ± 537	1008 ± 580	**< 0.001**	**< 0.001**	**0.004**
NLD T_max_	418 ± 316	564 ± 485	651 ± 330	*0.011*	*0.003*	0.060
NLD T_1/2_	1212 ± 592	1100 ± 648	1088 ± 579	0.470	0.255	0.550

Bold values indicate significant differences. Though representing significant differences, values in italics can only be taken into account in a restricted degree as the radioactive tracer can slightly reach NLDs in tearing eyes because of stenosis or obstruction

*n* number of patients/controls, *NLD* nasolacrimal duct, *p p* value

**Fig. 4:**
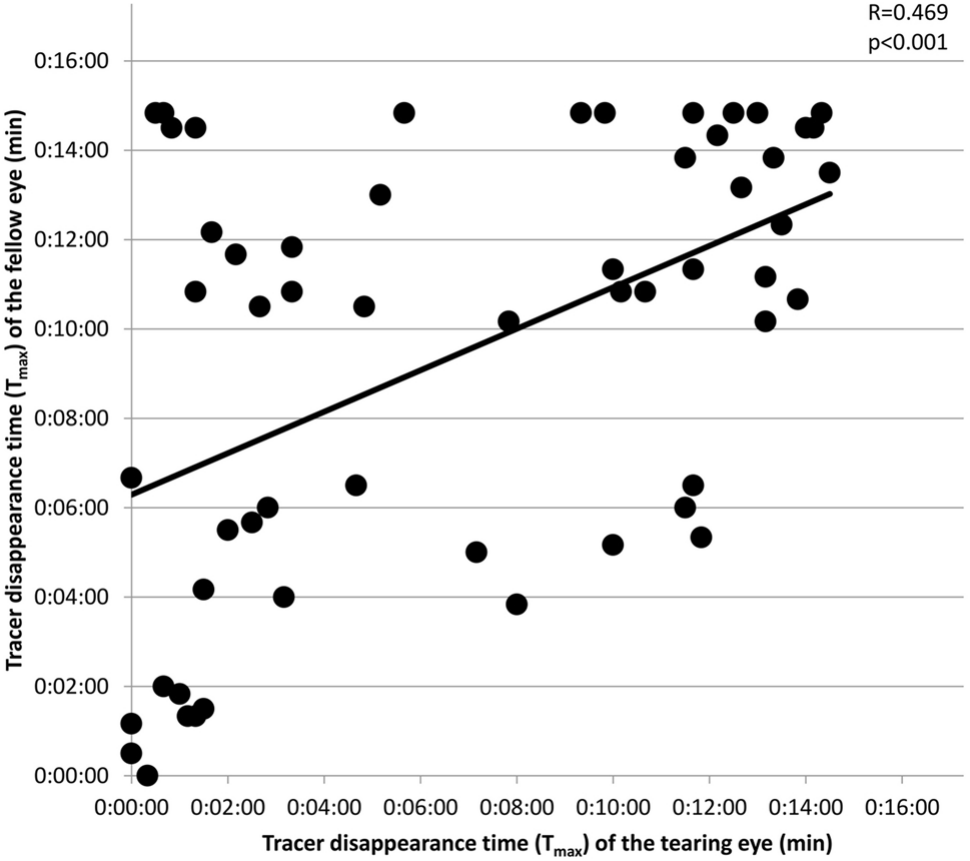
Correlation between maximum tracer disappearance time (T_max_) values of tearing and fellow eyes. *p p* value, *R* correlation coefficient

In general, the association between T_max_ values of the tearing and the fellow eyes was weak. Correlation coefficient was significant between T_max_ values of the LDS regions for the tearing and the fellow eye; the results are shown in Fig. 4. Concerning sensitivity, SPECT/CT proved to be the most sensitive method (87.72%), followed by CT-DCG (78.95%), and DSG (68.42%). While combining the techniques, the sensitivity values were as follows: DSG with SPECT/CT: 96.49%, DSG with CT-DCG: 92.98%, and SPECT/CT with CT-DCG: 94.73%, respectively. Specificity of DSG was found to be higher when compared to SPECT/CT technique. Data are represented in Table 2. The kappa coefficient used to test inter-rater reliability was 0.315 between CT-DCG and SPECT/CT with the difference being statistically significant (see Table 3), while it was − 0.020 between DSG and SPECT/CT, and 0.019 between DSG and CT-DCG with no statistical significance.

**Table 2:** Sensitivity and specificity of DSG, SPECT/CT, and CT-DCG procedures in the tearing, fellow, and control eyes

	Tearing eye (*n* = 57)	Fellow eye (*n* = 57)	Control eye (*n* = 32)	Sensitivity (%) (95% CI)	Specificity (%) (95% CI)	
					Fellow eye	Control eye
DSG positive	39 (68.42%)	23 (40.35%)	8 (25%)	68.42 (54.65–79.76)	59.65 (45.82–72.25)	75.0 (56.26–87.94)
SPECT/CT positive	50 (87.72%)	36 (63.16%)	–	87.72 (75.72–94.58)	36.84 (24.85–50.76)	–
CT-DCG positive	45 (78.95%)	–	–	78.95 (65.82–88.26)	–	–
DSG or SPECT/CT positive	55 (96.49%)	41 (71.93%)	–	96.49 (86.84–99.48)	28.07 (17.47–41.75)	–
DSG or CT-DCG positive	53 (92.98%)	–	–	92.98 (82.15–97.76)	–	–
SPECT/CT or CT-DCG positive	54 (94.73%)	–	–	94.73 (84.55–98.63)	–	–

*DSG* dacryoscintigraphy, *SPECT*/*CT* single-photon emission computed tomography/computed tomography, *CT*-*DCG* computed tomographic dacryocystography, *n* number of patients/controls, *CI* confidence interval

**Table 3:** Comparison of contrast CT and SPECT/CT cases based on inter-rater reliability

	SPECT/CT positive (*n* = 50)	SPECT/CT negative (*n* = 7)	Kappa coefficient	*p*
CT-DCG			0.315	0.012
Positive	42	3		
Negative	8	4		

*SPECT*/*CT* single-photon emission computed tomography/computed tomography, *CT*-*DCG* computed tomographic dacryocystography, *n* number of patients, *p p* value

## Discussion

The adequate volume and the appropriate, well-balanced composition of tears are indispensable for maintaining ocular surface health. Precorneal tear film has several essential functions including protecting the cornea from drying, contributing to the refractive power of the eye, or defending the structures inside the eye against infections. These roles are regulated by the lacrimal functional unit (LFU) that consists of the tear production system, i.e., the main and accessory lacrimal glands, the interconnecting innervations, fluid flow, tear evaporation, and last but not least the tear outflow through the LDS. Assessment of the LFU is crucial not only in consideration of diagnosing lacrimal apparatus disorders, but also in evaluating ocular surface processes and effectiveness of therapies in these lesions in follow-up periods. Both quantitative and qualitative alterations can occur related to LFU. Concerning the previous ones, epiphora is one of the most common ophthalmic symptoms. Its etiology can be broadly categorized into tear overproduction and inadequate drainage, and the latter can be put down to either anatomical or functional obstructions. The previous one, with a prevalence of 31.8–48.7%, can develop a blockage at any point along the LDS [11–13], while the latter one is termed for delayed or blocked tear outflow in the absence of mechanical obstruction in the LDS, and can be determined, from outermost to innermost, by dysfunction of the lacrimal pump, abnormal localization or blockage of the orifice, i.e., lacrimal puncta, and stenosis of the LDS [14]. To differentiate between stenosis and obstruction is indispensable since the former one can be treated by balloon catheter dilation or intubation, but the latter one, of which NLDO is the most frequent form being 4–5 times more prevalent in females [15] than in males, requires invasive surgical interventions [16, 17].

There are only scant objective techniques in the visualization and imaging of the LDS, and thus its abnormalities are mostly diagnosed via anamnestic data and a few clinical tests, which include tBUT, Schirmer, and Jones dye tests, syringing, and nasolacrimal probing [18, 19]. Among classic imaging modalities, X-ray, DCG, CT, or magnetic resonance imaging (MRI) can effectively assess obstruction; however, their success is much less in the evaluation of stenosis. On the contrary, DSG, being an objective and precise technique, provides real and accurate data about functional NLDO and supplies more detailed outcomes if partial obstruction occurs [20]. Hanna and co-workers analyzed the DSG results of 83 eyes of 67 patients, and though 55 LDSs were found to be patent by syringing, only 36 (65%) were found to be impaired by DSG [21]. The relevance of DSG is also supported by the fact that a great correspondence was reported between clinical assessment of epiphora and DSG in case of patent but non-functioning lacrimal systems [22].

DSG method is even capable of distinguishing localizations of LDS obstructions, and accordingly positive scintigrams were subdivided into prelacrimal sac, lacrimal sac/duct junction, and duct obstructions, and diverse incidences were described by different authors [20, 23–25]. Moreover, quantitative DSG, a measurement performed routinely in some departments [26], permits to quantify tear clearance rate and, based on time, activity curves can evaluate LFU globally [27].

It can be concluded that for the evaluation of the LFU under action, DSG is the most readily available, non-invasive method; however, it has some disadvantages. On the one hand, they are due to the fact that the lumen diameter of LDS is not constant but influenced by several drugs [28–30]. On the other hand, quantification can be elusive since it can provide little extra information if complete NLDO occurs. Moreover, to find an appropriate standardized point of reference related to an obstructed LDS comes up against difficulties since although the fellow, unaffected eye could be asymptomatic, stenosis or subclinical obstruction is often seen; as a consequence, it cannot always be used as a control [31]. In our study, the observation that fellow eyes T_max_ parameter was the highest and T_1/2_ quantity was higher compared to control eyes also supports this observation. Our theory about the reason why T_max_ value was the highest and T_1/2_ parameter was also increased for the region of nasolacrimal duct in case of control eyes is that ^99m^Tc-sodium pertechnetate can attach considerably to the mucosa or it is even absorbed by mucous membranes that cover the inner surface of the nasolacrimal drainage system. Our DSG data are in accordance with the literature [32].

Based on our outcomes, 8 out of the 32 control eyes DSG results were positive that were otherwise declared as clinically negative. As previously, several studies have found weak or no correlations between objective signs and subjective symptoms of dry eye disease (DED) [33, 34], further investigations are needed to verify possible LDS abnormalities or DED in these control eyes.

LDS can be involved even in a high-dose radioactive iodine (RAI) therapy that is applied against thyroid disorders. RAI is excreted in tears and actively accumulated in LDS and subsequently lacrimal gland impairment [35] as well as epiphora can develop since the low, but concentrated level of RAI induces inflammation that stimulates stenosis or obstruction [36]. Investigating RAI administration in a high number of patients, a cutoff level of 150 mCi was determined since toxicity and consecutive inflammation that leads to obstruction of LDS occurred in patients who received RAI doses above this level [37].

Although the application of a pinhole collimator camera during DSG [38] can be beneficial, it could be difficult to map the region of the eye with it because of the design of the collimator, since patients cannot fix their heads and not move. In our department, DSG using a pinhole collimator could have been performed only with patients in a lying position. Nevertheless, it is worth examining patients in near physiological conditions, namely in standing or sitting position as their complaints also occur when sitting or standing up. For cameras equipped with LEHR (low-energy high-resolution) collimators, patients can fix their chin and forehead in a special holder close enough to the detector, and also they are able to embrace the detector, so that potential movements will be rather negligible.

Thus, there is a general agreement across authors that because of some disadvantages DSG can provide only additional data to clinical evaluation, and nowadays it is mostly regarded as a supplementary method to evaluate tearing features which then may help specialists to select the most appropriate type of treatment. As a result of these considerations, other non-invasive or less invasive diagnostic techniques should be involved in the medical checkup of epiphoretic patients [39]. An efficient modality in diagnosing deviations of the LDS is DCG, and combining the two techniques sensitivity can be increased to 98% [23]. The discrepancy between the two techniques is mostly explained by the fact that during DCG a high-pressure contrast injection of fluid is administered into the LDS that can come through incomplete obstruction sites, while DSG imitates physiological tear flow, which makes it eligible to investigate functional epiphora insomuch as it is recommended as a first-line investigation in these cases [20]. Nevertheless, in the management of epiphoretic patients, DCG cannot be passed over since it is deemed to be the criterion standard for diagnostic imaging of LDS obstruction [40].

To the best of our knowledge, the first application of SPECT/CT for visualization of the LDS was used by Sakahara and et al. as early as 2007, who investigated the uptake of ^131^I in the LDS after radioiodine therapy [36]. Afterward simultaneous use of DSG and SPECT/CT combined with DCG was described by our team, where we identified the discrepancy between the passive DSG and actively executed DCG [10]. CT application was chosen as no other imaging facility can offer as distinct an anatomic detail of the bony LDS as CT. Anyway, CT-DCG can resolve pre-operative characterization of a possible occlusion by improving planar imaging of DSG, and this localization is required for a correct treatment planning. In our present survey, the kappa value measuring inter-rater reliability between different methods was found to be 0.315 between CT-DCG and SPECT/CT; and as the difference was statistically significant, it means that these two independent imaging techniques can lead to a matching decision.

## Conclusions

There are scant diagnostic conceptualizations and no standardized examinations for LDS abnormalities; consequently, evaluation of tearing patients is generally based on history, external ophthalmic physical examination, and straightforward office lacrimal tests enhanced when inevitable by nasal examination.

DSG is a widely employed effective modality in diagnosing abnormalities of the LDS which can provide relatively objective data on LDS impairments; however, when using a planar camera for imaging, this facility should be potentiated. Furthermore, surgical indication can be established only based on supported images, and additionally, precise localization of the obstruction can be merely determined by detailed imaging. The simultaneous application of DSG, SPECT/CT combined with CT-DCG can provide careful final results with high sensitivity.

The main advantage of the study is the measured control eyes involved in DSG since most of the surveys that deal with DSG of tearing patients quantify only the affected eyes. Another advantage is the wide range of imaging techniques that were compared. However, the main limitations of the study could be the relatively small sample size and lack of SPECT/CT and DCG modalities for control eyes due to ethical reasons.
